# Teachers’ SEL Identity (SEL-ID): An Intersection Between Teacher Identity and Social and Emotional Learning (SEL)

**DOI:** 10.3390/bs16010058

**Published:** 2025-12-30

**Authors:** Zehra Kaplan, Mine Göl-Güven

**Affiliations:** Program of Learning Sciences, Social Sciences Institute, Boğaziçi University, Istanbul 34342, Türkiye; mine.golguven@bogazici.edu.tr

**Keywords:** social and emotional learning identity (SEL-ID), teacher identity, social and emotional learning (SEL), prosocial classroom, early childhood education teachers

## Abstract

Although teacher identity and social–emotional learning (SEL) have been studied separately, little is known about how these constructs intersect in ways that explain why teachers’ social and emotional competence (SEC) does not always translate into classroom practice. This study introduces the construct of SEL identity (SEL-ID) as a potential missing piece in the current SEL frameworks by utilizing the teacher identity construct. This study seeks to describe SEL-ID, drawing on teachers’ reflections on lived experiences and their classroom practices. Using grounded theory, the data was collected through semi-structured interviews and classroom observations of 12 early childhood education teachers who were actively working with children aged from 3 to 5 in childcare centers established by a local municipality. As a result, the coding process revealed overlaps between teacher identity and SEL, as well as unique elements that go beyond the established SEL framework. Five interrelated components of SEL-ID resulted from the analysis process: (1) self-perception, (2) emotional literacy, (3) interpersonal relations, (4) participatory SEL, and (5) managerial expertise. These findings demonstrate that SEL-ID is not simply an extension of teacher identity or SEL but a construct that helps explain variations in teachers’ ability to enact SEL in practice. The researchers hope that this study will guide future studies to explore more into SEL-ID and its contribution to strengthening SEL practices in schools.

## 1. Introduction

Fifteen years ago, Jennings and Greenberg (2009) proposed the Prosocial Classroom Model, identifying teachers’ social and emotional competence (SEC) as one of the three key factors influencing children’s academic performance and overall well-being. Research guided by this model consistently shows that teachers’ social–emotional learning (SEL)-related practices contribute to supportive classroom environments, positive relationships, and stronger student engagement (Henriksen et al., 2025; McCormick et al., 2021; Poulou & Garner, 2025; Scheirlinckx et al., 2025; Schonert-Reichl, 2017). However, while teachers’ SEC is essential, it alone does not explain how teachers understand and practice SEL in their classrooms. Although several studies indicate that teachers’ SEC plays a crucial role in developing students’ SEL, some have not provided the underlying reasons for not obtaining the most benefit out of SEL programs (O’Brien et al., 2025; Wigelsworth et al., 2022). Nevertheless, evidence suggests that when SEL practices are applied inconsistently or without sustained commitment, their effectiveness may be significantly reduced, regardless of teachers’ recognition of their importance (Evans et al., 2015).

While SEL is recognized for promoting children’s self-awareness, emotion regulation, social skills, and overall well-being (CASEL, 2020; Durlak & Weissberg, 2011; Ekmekci & Serrano, 2022; Gimbert et al., 2023; Turan Bora & Akbaba Altun, 2025), the findings are somewhat contradictory. Some meta-analyses validate benefits while indicating small to moderate effect sizes, raising concerns regarding the long-term sustainability of these effects (Cipriano et al., 2023; Durlak et al., 2022; Lemberger-Truelove et al., 2025; Shi et al., 2025; Taylor et al., 2017). Critics assert that the conversation surrounding SEL may individualize structural issues by placing the burden of accountability on teachers and students, a perspective that has influenced the transformative SEL movement (Jagers et al., 2019). Such critiques underline that technical knowledge alone may be insufficient for teachers to integrate SEL meaningfully and consistently.

At this juncture, considering and exploring teacher identity from an SEL perspective is critical. Teacher identity, characterized as dynamic, complex, and context-dependent (Avraamidou, 2019; Beauchamp & Thomas, 2009), offers a framework to examine how teachers establish their professional identities, which may explain how SEL might be recognized, practiced, and integrated into teachers’ professional identities. Existing studies conceptualized multiple subject-specific identities, such as English (EFL) teacher identity (Varghese et al., 2005), science teacher identity (Beauchamp & Thomas, 2009), mathematics teacher identity (Hodgen & Askew, 2011), and music teacher identity (Ballantyne & Grootenboer, 2012). Building on this tradition, it can be argued that SEL-ID represents another form of teacher identity, one that emphasizes the integration of SEC into professional practice. Conceptualizing SEL-ID in this way not only aligns with existing discussions on the multiplicity of teacher identities but also highlights the unique role of SEL in shaping teachers’ self-perceptions, classroom practices, and relationships with students.

## 2. Conceptualization and Related Research

### 2.1. SEL in Education

SEL was introduced as a missing piece in the 1990’s to express the need in schools through paying attention to the “affective” component of teaching (Elias, 2003). For further definition, CASEL (2020) conceptualized SEL as a process by which individuals of all ages learn and apply knowledge, skills, and attitudes that help them build a positive sense of self, regulate their emotions, pursue personal and shared goals, demonstrate empathy, form and sustain supportive relationships, and make responsible choices. Within the framework of SEL, those competencies are conceptualized under five domains: self-awareness, self-management, social awareness, relationship skills, and responsible decision-making.

SEL has gained more and more attention since it promotes not only students’ social and emotional development but also their academic success (Durlak et al., 2011; Taylor et al., 2017). Research demonstrates that SEL interventions improve children’s academic performance while fostering prosocial behavior, emotional regulation, and psychological well-being (Cipriano et al., 2023; Durlak et al., 2022; Gol-Guven, 2017; Jones & Kahn, 2017; Zins et al., 2007). Meta-analyses validate minor to moderate positive effects on students’ academic and social–emotional outcomes (Durlak et al., 2022; Taylor et al., 2017). Furthermore, longitudinal studies indicate that SEL can have ongoing benefits, including less emotional distress, improved school attitudes, and enhanced social competence long after program implementation (Mahoney et al., 2021). As such, SEL has come to be recognized as a foundational element of high-quality education, one that supports both cognitive and non-cognitive domains of development (Jones & Bouffard, 2012; Schonert-Reichl, 2017). Rather than viewing schools solely as academic institutions, scholars and policymakers increasingly emphasize their role in promoting holistic development that prepares children for life beyond standardized outcomes (Jones & Kahn, 2017). This comprehensive perspective emphasizes how crucial it is to incorporate SEL into teaching methods to ensure that students’ overall well-being and academic development are supported by SEL.

Although SEL research reports positive outcomes, the findings remain somewhat paradoxical. Some research indicates significant outcomes, while others observe that the effect sizes are moderate and the long-term effect of these effects remains uncertain (Cipriano et al., 2023; Dymnicki et al., 2011; Hart et al., 2024). The impact of SEL on academic performance does not consistently meet the high expectations regarding its potential to enhance students’ academic achievement (McCormick et al., 2021). Additionally, the efficacy of SEL programs is not exclusively dependent on curriculum design. It mostly relies on the teacher’s ability to exemplify and integrate SEL principles into everyday classroom interactions (Jones & Bouffard, 2012). Teachers are vital in fostering an interpersonal and emotional environment necessary for SEL to be successful, stressing the significance of their own SEC as a crucial element of implementation and sustainability (Jennings et al., 2017). Moreover, the transformative SEL literature emphasizes the issue that traditional SEL, which largely focuses on individual skill development and behavior management, may unintentionally attribute systemic problems to individuals, therefore blaming teachers and students instead of confronting wider inequalities (Jagers et al., 2019; Ramirez et al., 2021). In contrast, transformative SEL adopts a more equity-oriented perspective by situating SEL within social, cultural, and structural contexts. Such criticisms emphasize that when SEL is solely conceptualized as a collection of individuals’ technical abilities, it may become superficial and complicated for teachers. Not having a common understanding of SEL and reducing SEL to a strategy may not be enough for consistent implementation. Rather, a strong sense of ownership and integration into teachers’ professional identities may be necessary.

Recent scholarship has begun to challenge the dominant portrayal of SEL as a largely individual endeavor. Critics contend that conventional SEL frameworks frequently center on behavioral compliance and personal regulation in ways that can mask deeper structural inequalities or constrain the scope for genuine agency among both students and teachers (Jagers et al., 2019; Ramirez et al., 2021). Transformative and equity-centered approaches, by contrast, insist that power dynamics, agency, belonging, and meaningful participation must occupy the foreground of any robust SEL implementation. Rather than treating emotional and relational skills as ends in themselves, these perspectives reorient the work toward cultivating the capacity of children and adults alike to analyze, decide, and intervene in the social worlds they inhabit.

### 2.2. Teacher SEC and Learning Environment

Teachers’ SEC is an important factor for both their own quality of life and classroom practices, their relationships with children, and children’s well-being (Fitzgerald et al., 2022). Jennings and Greenberg (2009) proposed the Prosocial Classroom Model, which focuses on the central role of teachers’ SEC on classroom climate and student outcomes. Teachers with high SEC can effectively identify and regulate their own emotional states and cognition, which prevents emotional burnout and enables them to respond effectively to students’ needs, including both academic and social–emotional, by providing supportive learning environments (Hemi & Kasperski, 2023; Jennings et al., 2017; Jennings et al., 2019; McCormick et al., 2021; Schonert-Reichl, 2019). In addition, research indicates that teachers’ SEC influence their effective classroom management strategies, the relationships they establish with students, which predict better achievement and engagement of students (Cappella et al., 2012; Hamre & Pianta, 2001), and their ability to implement SEL skills (Jennings & Greenberg, 2009; Jennings et al., 2021; Schonert-Reichl, 2017). Along with outcomes related to the classroom, socially competent teachers feel more fulfilled and derive greater enjoyment and satisfaction from teaching (Goddard et al., 2000). Such competencies enhance the quality of teachers’ interactions with students and promote the decrease in maladaptive behaviors (Humphries et al., 2018; Perry & Ball, 2008; Roeser et al., 2012).

Moreover, SEL-focused intervention (e.g., Lions Quest) helps teachers increase their perceived competence in relation to SEL significantly (Talvio et al., 2021). Yet, many teachers, even if their theoretical understanding of SEL is strong, have a limited number of practical strategies, suggesting that knowledge is not always transferred to practice (Ferreira et al., 2025). In alignment with this, research on Lions Quest in Turkiye revealed that while teachers demonstrated favorable attitudes toward SEL, they frequently did not fully embrace responsibility for it, perceiving SEL instead as a developmental process, a parental duty, or a phenomenon that naturally arises as children adapt to the school environment (Gol-Guven, 2016). Collectively, these studies highlight a gap between teachers’ attitudes and actual ownership of SEL in classroom practice.

Although noteworthy, evidence indicates that SEC, while essential, may not adequately account for variability in teacher classroom practices. Research indicates that teachers with similar levels of SEC present variability in their capacity to implement effective SEL practices, influenced by contextual and personal factors including school culture, administrative support, and teacher characteristics (Thierry et al., 2022; Ulla & Poom-Valickis, 2023). Even though these external factors constrain teachers’ SEL practices, they alone do not sufficiently explain the lack of deep integration of SEL into everyday teaching. Research on teacher identity formation illustrates that pre-service periods are critical for developing a commitment to learning (Young et al., 2022). Moreover, the majority of teachers have no formal training in SEL, leaving them insufficiently equipped to integrate these competencies into their teaching (Duane et al., 2025; Jennings & Greenberg, 2009). Teachers may show a tendency to prioritize emotional survival, focusing primarily on getting through the day by managing immediate stressors and avoiding becoming emotionally overwhelmed rather than engaging in proactive, pedagogically grounded SEL practices, and this emotional survival tendency can increase stress and burnout without professional development opportunities (Davis & Park, 2025).

While SEC significantly affects the quality of classroom interactions, the majority of studies emphasize teachers’ knowledge and skills, neglecting to examine how these competencies are transmitted and exhibited in everyday practice (Jennings et al., 2017; Schonert-Reichl, 2017). This shows a necessity to progress beyond technical skills towards frameworks that encompass teachers’ sense of ownership and professional integration of SEL.

### 2.3. Teacher Identity

Teacher identity is a dynamic and ongoing process related to how a person perceives themselves as a teacher and how they are perceived by others (Beauchamp & Thomas, 2009; Kaplan & Garner, 2017; Olsen, 2016; Starr et al., 2006). This process involves the individual exploring their own values, beliefs, and roles, as well as understanding the values and norms of the teaching profession and integrating their personal identity with their professional identity (Beauchamp & Thomas, 2009; Beijaard et al., 2004; Flores & Day, 2006; Korthagen, 2004; Olsen, 2016; Schepens et al., 2009). Teacher identity is a multidimensional construct, including the cognitive and emotional aspects of teachers (Avraamidou, 2014; Kelchtermans & Deketelaere, 2016; Nias, 1996; Zembylas, 2005, 2007), and has an impact on teachers’ sense of purpose, self-efficacy, motivation, commitment, and job satisfaction (Beijaard et al., 2004; Day et al., 2006). It is conceptualized as the combination of self-image, motivation, commitment, task perception, self-efficacy, and job satisfaction (Hanna et al., 2019). The development of teacher identity begins with the pre-existing beliefs students hold when they join teaching programs and demonstrates significant growth through lessons and teaching practices (Friesen & Besley, 2013). The educational context, prior experiences, and individual backgrounds are also fundamental factors that influence teacher identity (Beijaard et al., 2000; Day et al., 2006). Emotional support received from collaborative environments and the positive relationships established contribute to the formation of teachers’ professional self-concept by increasing their sense of belonging and motivation to the profession (Kelchtermans & Deketelaere, 2016).

Researchers emphasize that identity is specific to both the subject and the context by demonstrating various investigations of the identities, such as language teachers, science teachers, mathematics teachers, and music teachers (Ballantyne & Grootenboer, 2012; Beauchamp & Thomas, 2009; Hodgen & Askew, 2011; Varghese et al., 2005). These studies demonstrate that identity manifests in various forms based on teaching areas, circumstances, and expectations, emphasizing that identity is not solely cognitive but also profoundly emotional and relational (Zembylas, 2005, 2007). Recent evaluations indicate that studies on teacher identity have broadened to encompass intersections with social justice, agency, and equity (Andrews et al., 2018) that are also emphasized in transformative SEL (Jagers et al., 2019).

Establishing a clearer conceptual bridge between teacher identity and SEL requires identifying only the dimensions that meaningfully intersect across these two bodies of literature (CASEL, 2020; Hanna et al., 2020). There is an intersection between the components of teacher identity and SEC. For example, self-image, which refers to one’s feeling related to a sense of belonging to a community, aligns with self-awareness involving integration of personal and social identities. Another intersection is between self-efficacy and self-management. Self-efficacy is a belief related to the capabilities to perform teaching-related activities linked to self-management involving planning and organization skills. In addition, task perception is one’s belief related to priorities or duties linked to social awareness, and teachers’ understanding of their role depends on how they perceive children’s needs, family contexts, and social–cultural dynamics.

Although identity is recognized as essential for understanding the way in which teachers fulfill their professional roles, the current SEL framework appears to contain a missing piece: it underlines teachers’ SEC yet does not explain why strong SEC does not always lead to meaningful SEL practices in classrooms. In this study, teacher identity is used as a conceptual entry to introduce a new construct. This encourages an inquiry into whether defining SEL-ID as a particular variant of teacher identity might reveal the reasons for the limited reflections of SEC in classroom activities. SEL-ID stresses the importance of teachers’ ownership of SEL, conceptualizing it not merely as a collection of skills but as integral to their professional identity.

### 2.4. The Purpose of the Study

This study aims to introduce and conceptualize a new form of teacher identity, SEL-ID, to the field of SEL and understand how SEL-ID is established and reflected in SEL classroom practices. Therefore, this study focused on the following research question: “What constitutes SEL identity for early childhood teachers, as revealed through their reflections and practices?”

## 3. Methodology

### 3.1. Participants

The participants in this study were all female teachers with diverse levels of teaching experience; specifically, four teachers with 0 to 3 years, five teachers with 4 to 6 years, one teacher with 7 to 9 years, one teacher with 10 to 12 years, and one teacher with more than 16 years of experience. Two teachers had graduated with a 2-year degree and a Master’s degree, whereas ten teachers had Bachelor’s degrees. Table 1 shows the pseudonyms of the participant teachers, their educational levels, and their years of experience.

### 3.2. Research Design and Procedure

A grounded theory design was utilized to inductively formulate a data-driven conceptualization in order to address the emergence of SEL-ID (Charmaz, 2014; Corbin & Strauss, 2014). Given the interpretive nature of grounded theory, the aim of this study was not to achieve statistical generalizability but to generate a context-grounded conceptual understanding of SEL-ID. Consistent with qualitative research traditions, the study sought depth, meaning, and theoretical explanation rather than population-level inference. In this sense, the value of the findings rests on their analytical and naturalistic generalizability that shows how the conceptual categories may resonate with similar educational contexts and how the theoretical insights may be transferred or adapted, rather than generalized in a statistical sense (Smith, 2018; Carminati, 2018). This approach aligns with qualitative methodology, which prioritizes conceptual robustness and contextual richness over representativeness. Early childhood education (ECE) teachers were deliberately chosen because of the frequent, relational, and emotionally intensive interactions they have with children in early years environments, which make SEL implementation and teacher identity particularly significant (Hamre & Pianta, 2001; Pianta, 2001). Ethical approval was secured from the university of interest before recruitment took place.

The study took place during a planned teacher education program involving ECE teachers employed in ECE Centers established by a metropolitan municipality. Teachers were informed about the study, and their voluntary participation was sought. This study was guided by the Prosocial Classroom Model (Jennings & Greenberg, 2009), which makes the case that SEC influences classroom dynamics primarily through the quality of teacher–student relationships, which in turn impacts student outcomes. In addition, the studies have shown a connection between teacher identity and student–teacher relationships (Beauchamp & Thomas, 2009; Day et al., 2006). Student–teacher relationships reflect the quality of interaction practices and student outcomes (Pianta et al., 2012). With this logic, we used purposeful sampling. This also allowed us to diversify teacher selection by having this study’s main concepts, including identity (who I am as a teacher), social and emotional competencies (the competencies I have), and student–teacher relationship (the relational dynamics of SEL skills). Four profiles were used: (1) teachers who received high scores in all three scales, (2) teachers who received low scores in all three scales, (3) teachers who received high scores on competence and relationship but low scores on identity, and (4) teachers who received high scores on identity and competence but low scores on relationships. The four profiles were developed to capture diverse combinations of teacher identity, SEC, and student–teacher relationship quality. This variation allowed for a more nuanced exploration of how SEL-ID takes shape across different teacher characteristics, rather than limiting the analysis to a homogeneous sample.

Accordingly, participants filled in a digital survey package consisting of three scales that measure teacher identity, SEL, and student–teacher relationships. The scales are the Social–Emotional Competence Teacher Rating Scale (Tom, 2012), the Teacher Identity Scale (Hanna et al., 2020), and the Student–Teacher Relationship Scale (STRS) (Pianta, 2001). The purpose of administering these scales was not to analyze quantitative outcomes but to identify teachers with diverse profiles in terms of identity, SEC, and relational competencies. After calculating the scores obtained from the survey package, 51 teachers were classified into four profiles: Four teachers were not reached due to the wrong email and phone information, eight teachers were excluded since they did not have a stable class, two teachers were excluded since they obtained an administrative position, eleven teachers did not return the study invitation through email or phone, and fourteen teachers opted out due to the time constraints and workload at their centers. In the end, 12 teachers agreed to participate in the study. Within the 4-month period, each teacher participated in three different interviews, each lasting about 45 min, focusing on different aspects of their identity, SEL, and classroom practices. In addition, they were observed on three separate half-day occasions. Conducting multiple interviews and observations ensured data richness and representativeness, as a single encounter would not have adequately captured the complexity of teachers’ practices and perspectives. Following the methodology of grounded theory, data collection and analysis were conducted concurrently in an iterative process. Initial coding was followed by focused coding, exploring patterns and the development of categories that constituted SEL identity.

### 3.3. Data Collection

The current study used semi-structured interviews and classroom observations to conceptualize SEL-ID through teachers’ beliefs, knowledge, practices, and lived narratives. Details regarding interview and observation procedures, including duration and guiding questions, are provided in Table 2.

#### 3.3.1. Semi-Structured Interviews

Data was collected through semi-structured interviews with 12 ECE teachers. Each teacher participated in three individual interviews, conducted face-to-face, with each session lasting approximately 45 min. All interviews were audio-recorded with participants’ consent and later transcribed for analysis. The interviews followed a flexible protocol that allowed adaptation across sessions in line with grounded theory methodology.

The first interview focused on teacher narratives and professional identity, with questions such as “How did you decide to become a teacher?” and “How do you perceive your role as a teacher?” The second interview centered on teachers’ beliefs, emotions, and knowledge related to SEL, with questions such as “How do your emotions play a role in your teaching?” and “Can you share moments when your emotions have influenced your practice?” The third interview shifted toward classroom practice, asking questions such as “As a teacher, what are your priorities in the classroom or in education?” and “What strategies do you use to support SEL?”

Observations from the classroom were occasionally incorporated into the interviews to provide clarity or encourage further reflection on the observed behaviors. This iterative methodology, in which emerging insights guided future interviews, facilitated a deeper comprehension of teachers’ SEL-related behaviors.

#### 3.3.2. Classroom Observations

Classroom observations were conducted with the same 12 teachers prior to and in parallel with the interviews. Each teacher was observed three times, with each observation lasting approximately half a day. Observations were guided by the Classroom Assessment Scoring System (CLASS) framework (Pianta et al., 2008), which provided structured attention to classroom practices. Emphasis was placed on teachers’ emotional expressiveness, sensitivity to children’s needs, strategies used for conflict resolution and behavior management, and integration of SEL into daily routines.

Observation sessions were documented through detailed notes that included chronological accounts of classroom activities, teacher–child interactions, and contextual factors. Notes also captured aspects such as changes in classroom dynamics, teacher mood, and interruptions in daily routines. These observations served both as a primary source of data on classroom practices and as a means of triangulating interview findings, allowing the study to assess how teachers’ self-reported perspectives aligned with their enacted practices in real classroom settings.

### 3.4. Data Analysis

After transcribing and preparing the data, a data cleansing process was undertaken to ensure accuracy and consistency. This involved checking transcripts against audio recordings, correcting transcription errors, and removing incomplete or irrelevant responses. Such cleaning was necessary to enhance the trustworthiness of the data set before analysis. MAXQDA 24 software was used to organize and code the data systematically. The analysis followed Charmaz’s (2014) grounded theory approach. Following this step, the data were read multiple times to increase familiarity, after which initial line-by-line coding was conducted. In axial coding, after the open coding process, initial codes were compared and clustered around the concepts of teacher identity and the SEL framework. Codes were then constantly compared with one another and with sensitizing concepts from the teacher identity and SEL literatures. This process revealed both overlaps (e.g., commitment and self-awareness) and elements that did not directly fit within existing frameworks (e.g., child participation in decision making, fostering SEL). As an example, while comparing data iteratively with both emerging categories and existing constructs, new categories were generated that went beyond teacher identity and SEL alone. For example, while self-perception and emotional literacy reflected intersections with established frameworks, categories such as participatory SEL and managerial expertise emerged from the data, distinct from SEL or teacher identity while at the same time reflecting the synthesis of both frameworks in practice. In this way, the grounded theory process of constant comparison facilitated concept development, leading to the identification of five interrelated components of SEL identity. Through focused coding, these similarities and divergences were clustered into higher-order categories. Table 3 provides the information for coding by presenting the data and its relation to different levels of coding. It presents the step-by-step analytical progression from data excerpts to SEL-ID themes. Throughout the iterative coding process, the emergence of new codes was monitored to determine saturation. Drawing on Rahimi and Khatooni’s (2024) typology of saturation, code/thematic saturation was achieved. Although three interviews and three observations had been planned for each teacher from the beginning, new codes continued to emerge until approximately the twentieth interview. After this point, no additional codes or themes appeared in the data, indicating that code/thematic saturation had been reached. This stabilization of the coding structure provided confidence that the conceptual categories underpinning SEL-ID were sufficiently rich, comprehensive, and grounded in the data.

Through constant comparison, categories were developed and refined, some of which extended beyond existing SEL and teacher identity frameworks. These will be detailed in the findings section. Throughout this process, the authors collaborated closely in using constant comparison and identifying emerging concepts. They engaged in regular discussions to decide on the consistency of codes and to ensure that the data provided evidence for the identified concepts. Disagreements were resolved through dialogue until consensus was reached, which strengthened the credibility and coherence of the coding process. To increase robustness, a codebook was created and was iteratively refined as new data segments were compared with existing codes (Nowell et al., 2017). This made it clear how codes were defined and used. Also, memos were written during the whole process to keep track of coding choices, new interpretations, and possible other explanations. This made for a clear audit trail. These strategies supported dependability and allowed the researchers to verify that conceptual patterns were grounded in the data rather than researcher expectations.

## 4. Findings and Discussion

**Figure 1:**
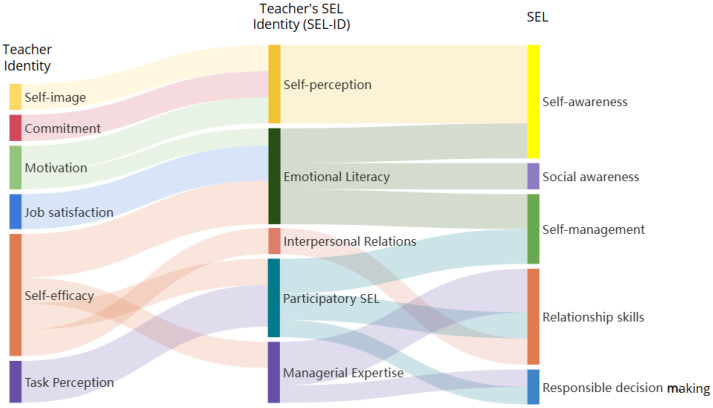
The interconnection of teachers’ identity and teachers’ SEL identity and SEL.

The data analysis demonstrated that the teacher’s SEL-ID is a complex construct that incorporates teacher identity and SEC. Figure 1 shows how teachers’ identity and SEL are interconnected, forming the SEL identity of teachers. The subconstructs of teacher identity are presented on the left side of the figure, whereas SEC is positioned on the right side. At the core of the analysis, five components of SEL-ID were identified: (a) self-perception, (b) emotional literacy, (c) interpersonal relations, (d) participatory SEL, and (e) managerial expertise. The following paragraphs provide the details of each of the five components supported with the data. The first three components, self-perception, emotional literacy, and interpersonal relations, emerged predominantly from interviews, whereas the latter two components, participatory SEL and managerial expertise, were primarily derived from classroom observations. This distinction highlights how different data sources provide unique insights into teachers’ SEL-ID.

### 4.1. Self-Perception

One of the fundamental elements of teachers’ SEL-ID is self-perception, which is defined as the grasp of their own identity, self-awareness, and self-image as influenced by their growth mindset, identity, and self-knowledge. It includes their capacity to identify their feelings, values, and strengths (e.g., recognizing emotions, such as frustration or pride, valuing honesty, or acknowledging their strengths) while establishing a sense of connection and sustaining internal drive for both career and personal development. Furthermore, career commitment emphasizes their devotion to long-term professional objectives, and affective commitment shows their emotional attachment to and identification with their teaching responsibilities. Teachers in the study expressed their motivation to grow and their recognition of personal strengths and weaknesses. Buse described how her personal interests outside teaching were closely tied to her sense of professional growth. She explained that by engaging more deeply with her interests, she hoped to enrich both her own well-being and her profession. As she mentioned, “I want to concentrate more on music and other art forms in my hobbies to enrich my job.” (Buse, interview 1.)

Another example from Ada shows teachers’ awareness related to self-knowledge, including personal standards. Her personal standards showed how she could not tolerate leaving tasks unfinished. Completing a task was, for her, essential not only for personal satisfaction but also for maintaining a positive sense of self.

“… *I really dislike leaving any task unfinished. I cannot stand it when something is left hanging like a stone in the air; I feel I must complete it. My intrinsic motivation comes from this-being able to like myself requires that I finish what I start. For example, if I clean a table, I might even wipe it twice just so I can say, ‘Great, it’s really done well*.”

(Ada, interview 1.)

Her narrative further illustrated a competitive spirit and a drive for continuous renewal. She emphasized that persistence and productivity were central to her identity.

*“I have a competitive spirit. I never give up. I try to do the best I can with the tasks I am given. I enjoy pursuing resources that will renew me, taking on responsibilities in this regard, putting something out there… I enjoy getting my hands dirty and being productive.”*

(Ada, interview 1.)

These expressions showed how self-perception captures both awareness of personal standards and a growth-oriented mindset. In the conceptualization of self-perception, it is more than mere self-knowledge as a fixed attribute; it constitutes the emotional and fundamental basis of SEL identity and influences how educators interpret, implement, and promote SEL practices. The passage from Ada shows how her personal standards, intrinsic motivation, and persistence are deeper parts of her identity that affect her emotional labor, professional commitment, and presence in the classroom. These types of self-awareness, such as competitiveness, productivity, and the desire to finish tasks, do not directly reflect SEL practice, but they do help explain how teachers deal with relational, emotional, and ethical demands in the classroom. In this context, self-perception serves as the motivational and identity-based foundation from which SEL practices develop.

Self-perception was not only reflected in teachers’ growth mindset and persistence but also in their ability to recognize personal limitations. For instance, Deniz expressed difficulty in achieving fairness, acknowledging that she often struggled to find balance in life. She stated *“I am not good at behaving in a fair way; I can’t find the balance.”* (Deniz, interview 2.) Unlike teachers who highlighted their productivity, Deniz’s statement illustrates how self-knowledge also involves awareness of shortcomings.

These examples illustrate teachers’ intrinsic motivation for growth and their awareness of self in the teaching role, both of which are concepts that appear in teacher identity and SEL frameworks. Consistent with extensive teacher identity research (Beauchamp & Thomas, 2009; Hanna et al., 2019; Rodgers & Scott, 2008), self-perception is linked to coherence, reflection, and purpose in professional roles. This aligns with one of the components in the CASEL framework, which is self-awareness, and also aligns with self-determination theory, which highlights autonomy, competence, and relatedness as fundamental drives of intrinsic motivation (Ryan & Deci, 2020).

These components work together to strengthen their sense of self as teachers, as does their global teacher identity and feeling of community. The finding contributes to the existing literature by underscoring the significance of teachers’ affective commitment and career commitment in shaping self-perception within SEL-ID, showing that SEL competencies are most effective when incorporated into teachers’ professional self-concept. This points out the need for supporting reflective practices in professional development that improve both abilities and ongoing awareness of identity.

Self-perception and self-image further exemplify the intersection between SEL identity and teacher identity. Professional identity conceptualizations underscore coherence, reflection, and purpose within extensive social and professional frameworks (Beauchamp & Thomas, 2009; Rodgers & Scott, 2008). These characteristics closely correspond with SEC, including self-awareness and self-reflection, which empower teachers to identify their values and strengths. A robust global teacher identity encompasses a sense of belonging to a community (Gee, 2000), which is recognized to enhance teachers’ motivation, well-being, and efficacy (Lambert et al., 2013; Schonert-Reichl, 2017).

Commitment, a subconstruct of teacher identity, is also fundamental to SEL identity. Teachers who view teaching as integral to their identity are more inclined to maintain a long-term commitment to the profession and demonstrate robust emotional connections to their positions (Canrinus et al., 2012; Wang et al., 2024). This viewpoint posits that self-perception fosters both emotional and professional commitment, strengthening teachers’ emotional attachment and professional resilience (Yağan et al., 2022).

### 4.2. Emotional Literacy

Emotional literacy, the second component of SEL-ID, can be conceptualized by integrating planning and emotional knowledge (e.g., strategic use of planning and goal setting to manage emotional demands and align actions with professional objectives), recognizing and managing emotions (e.g., the ability to recognize and regulate emotions to maintain a positive and productive teaching environment), and understanding how emotions affect behavior (e.g., awareness of how emotions influence decisions and interactions with students, colleagues, and parents). The following statements illustrate how teachers reflect and manage their emotions. Deniz, for example, openly acknowledged the emotional strain she experienced, reflecting on moments of exhaustion and self-doubt. *“I feel so inadequate, so tired, I wonder if I can do it.”* (Deniz, interview 1.) Her story illustrates how emotional awareness was closely linked to her professional confidence. In addition, Buse underlies how teacher emotion affects interaction in the classroom by saying “*We experienced a sad situation that day. When we experience sadness, we can feel that difference in the classroom.”* (Buse, interview 1.) Hale described concrete strategies for managing stress she experienced during duty days, which often changed her interactions with children adversely, yet she actively sought ways to cope.

*“On days when I’m on duty, I get very tense. I get very irritable, and this energy rubs off on the children… art… I work with watercolors. Adrenaline music relaxes me, I don’t know. It helps clear my mind, I love it.”*

(Hale, interview 1.)

Hale’s narrative demonstrates how art and music can help teachers to regulate the emotion and stress caused by the workload. Gonca reflected on the challenge of anger, admitting that her first reaction was to raise her voice, but she also described the strategies she relied on when support was available.

*“I’ve never been angry before. I don’t know what to do. I raise my voice, yes, that’s probably the first thing a teacher does, raising voice. Sometimes friends say, “Your voice is loud,” but yes, sometimes you say, “When you get angry, what do you do?” Well, your voice gets louder. So, if you can’t leave the classroom at that moment and there’s nothing else you can do, your voice gets louder-that’s the first thing happens. If I have a partner teacher in the class and I can go outside, I can take a breath, talk to my family, have something warm to drink.”*

(Gonca, interview 2.)

Finally, Ada also described how she processed emotions through extended reflection before reaching regulation. She explained that she could not immediately let go of emotionally challenging experiences but instead needed time and space to think through them repeatedly until she could reframe them.

*“I can’t just let go off of things immediately; I need to go home, lie down, and think more intensely. Sometimes it costs me a whole night. Certain issues keep me turning from side to side, rethinking and complicating them, then resolving them again. Later I motivate myself, telling myself, ‘This is not as big a problem as I thought.’ In time, everything goes back to normal.”*

Her account illustrates the role of rumination and cognitive reframing as strategies for emotional regulation (Ada, interview 2).

In the teachers’ quotes, themes such as emotional awareness, emotion regulation, and intentional planning for growth emerged. These patterns aligned with SEL competencies (self-awareness and self-management) and overlapped with teacher identity constructs, such as commitment to professional growth. Emotion regulation is a fundamental element of SEL and teaching effectiveness, as shown in the studies of Brackett et al. (2010) and Jennings and Greenberg (2009). In addition, regarding teacher identity studies, emotions are not perceived as simply personal traits but an integral part of how teachers build up their professional selves (Schunk & Pajares, 2009; Sutton & Wheatley, 2003). Zembylas (2005, 2007) argues that emotions play a role in shaping teacher identity and pedagogical choices. Kelchtermans and Deketelaere (2016) emphasize that the emotional involvement of teachers is essential for identity development and professional learning. These studies align with the emotional literacy component, which posits that emotional literacy is a part of identity that explains teachers’ emotional awareness and regulation, both as individuals and as professionals within school contexts.

Emotional literacy is also intertwined with teachers’ lived experiences, as previous emotional experiences influence their professional identity. Teachers’ narratives indicate that both positive and unpleasant school experiences significantly influence teachers’ perceptions of emotions and relationships in teaching (Avraamidou, 2019; Bächler & Salas, 2021). Surprisingly, teachers with negative past experiences in this study reported that they did not prefer to be a teacher as they had in the past, and they tried to be a better teacher. Beyond lived experiences, emotional literacy was linked to job satisfaction such that teachers possessing elevated levels of SEC are more successful at stress management, fostering positive interactions, and maintaining well-being, leading to diminished burnout and enhanced professional satisfaction (Brackett et al., 2010; Jennings & Greenberg, 2009; Wang et al., 2024). Another connection lies in a part of self-efficacy named affective self-efficacy, defined as the capacity to manage emotional responses (Bandura et al., 2003), which is intricately linked to SEC. Contrary to Hanna et al.’s (2020) teacher identity study, emotions were incorporated in SEL-ID while acknowledging the role of emotions on teachers as individuals and as professionals, which aligns with Hargreaves’s (1998) definition of emotional labor for the teaching profession.

### 4.3. Interpersonal Relations

Building relationships (e.g., the ability to establish positive and trustworthy relationships with students, colleagues, and the school community), using empathy in communication (e.g., the effective use of empathy for clear, empathetic, and respectful communication in professional interactions), and providing collegial support (e.g., active engagement and contribution to a supportive and collaborative network with colleagues) are all examples of interpersonal relations, which is the third component of SEL-ID. Here are a few data points pertaining to interpersonal relations. For example, Gonca explained how much she valued friendships within her school context, noting that having supportive colleagues was a key source of motivation. *“I mean, I think it’s important that we have good friends here, I mean, I think the important friendship motivates you.”* (Gonca, interview 1.)

Similarly, Beril discussed the culture of collaboration in her workplace, emphasizing how colleagues protected and supported each other in times of need. *“I see it all the time. If there’s a problem, everyone covers each other’s backs immediately.”* (Beril, interview 1.) This perspective was also supported by classroom observations. For instance, during observation 3, it was noted that the support teacher in Beril’s classroom was pregnant and occasionally required breaks. Whenever such a need arose, Beril encouraged her colleague to leave the classroom to take care of herself, reassuring her that the class would be managed in her absence. This example illustrates collegial support enacted not only in professional but also in emotional terms, reflecting the dual nature of support that extends beyond instructional responsibilities.

These examples illustrated building relationships and collegial support, which are similar to teacher identity’s self-efficacy and relationship skills in SEL. However, unlike broader teacher identity frameworks, teachers in this study highlighted collegial support for both the emotional and the professional as a distinct and defining elements of their identity. The interpersonal relation component shows similarity with the relationship skills of CASEL’s framework (CASEL, 2020), whereas it links with self-efficacy and collaboration studies in teacher identity (Brouwers et al., 2001; Klassen et al., 2011). Healthy teacher–student interactions forecast favorable classroom outcomes (Hamre & Pianta, 2001), whereas collegial connections enhance teacher motivation and job satisfaction (Lambert et al., 2013). This study uniquely highlights collegial support as being both emotional and professional. While the SEL frameworks underline teacher–student relationships, the findings of the current study indicated that SEL-ID includes teacher interactions with peers as essential for maintaining SEL in professional life.

### 4.4. Participatory SEL

The fourth component of SEL-ID is participatory SEL, which includes the cultivation of SEL skills (e.g., explicit instruction in SEL skills such as emotion regulation, goal setting, and effective collaboration), the enhancement of self-management (e.g., providing students with strategies to regulate emotions and behaviors, thereby facilitating productive group work), the promotion of student participation (e.g., fostering student voice and active engagement in classroom decision-making), and the perception of education (e.g., viewing education as a means for personal and community development, with teaching practices aligned to these principles). As this component was primarily observed in classroom practices, many samples were drawn from observation notes and subsequent teacher reflections. For instance, during one observation, Beril provided two options to the students, asking whether they wanted to go outside or stay in the classroom. After documenting this moment in field notes, we asked her about the purpose and meaning of doing so. She explained that

*“In terms of prioritizing, I usually put children to a vote when we experience such things. I usually vote on whether we should go to the playground or the park, whichever has more fingers, whichever has more applause, you know, I usually prefer this way, but if both experiences are desired, if there is an experience in the middle and it is beautiful, I will definitely do both.”*

(Beril, interview 3.)

This example illustrates children’s participation in classroom decision-making, a practice that reflects the teacher’s sense of self-efficacy and her confidence in facilitating shared choices, while also modeling SEL skills that extend beyond those explicitly outlined in the SEL framework. As seen in the example, SEL was not only taught explicitly but also modeled through everyday classroom routines by giving space for children’s involvement. This links with transformative SEL, which underlies the importance of practices exceeding individual skill development and improving equity, agency, and collective responsibility (Jagers et al., 2019). Studies in the SEL literature suggest that integrating SEL into the classroom is more influential when allowing student voice and making decisions together in learning environments (Cipriano et al., 2023; Mahoney et al., 2021).

A second set of practices emerged not from decision-making activities but from teachers’ use of instructional strategies that supported SEL. For example, during an observation of Senem’s classroom, it was noted that following lunch, when students had difficulty concentrating and displayed increased physical restlessness, the teacher initiated a yoga circle. Although children were not involved in deciding whether to do yoga, they actively participated in a structured activity designed to help them manage emotional and physical states. This aligns with participatory SEL in the sense that it supports students’ self-management and co-regulation through teacher-facilitated SEL-oriented routines. Children participated with interest, and those who were particularly active engaged in breathing exercises that helped them settle into a calmer state. This practice illustrates how participatory SEL can enhance students’ self-management by offering concrete strategies for emotion regulation and behavioral control, integrated seamlessly into daily routines. When teachers translate their beliefs about student participation into concrete classroom practices, these participatory approaches help reduce problem behaviors and strengthen students’ sense of belonging (Jones & Kahn, 2017; Weissberg et al., 2015).

In the classroom observation of Selin, she adapted her classroom routine when the children could not go outside due to rainy weather. She suggested, *“Let’s get the yoga cards and do some movement together,”* and led the children in yoga activities. The students participated enthusiastically, engaging in stretching and balancing exercises. When asked afterwards about her purpose in introducing yoga, Selin explained that it was not primarily intended to teach self-management but rather to provide physical exercise and variation. *“The children couldn’t go out to the garden, so we did it as exercise, as movement. Sometimes we do it like sports; it creates variety.”* Her stated intention was not explicitly to foster SEL skills, which illustrates intentional variation among teachers in how they interpret and integrate SEL-related practices into their daily routines.

Beyond the SEL literature, it may be useful to consider task perception that is asserted by teacher identity. Task perception revealed more shared understanding and was defined as teachers’ perceptions of their professional obligations (Hanna et al., 2019; Richter et al., 2021), encompassing beliefs regarding whether teaching entails the transmission of knowledge or the promotion of larger educational objectives such as autonomy, responsibility, and citizenship. Task perception is shaped by the values and commitments of teachers (Avraamidou, 2019; Beauchamp & Thomas, 2009). Teachers who embrace a facilitator approach are more inclined to employ participatory SEL techniques, fostering student agency, decision-making, and critical thinking (Jones et al., 2013). Teachers who perceive their duty as promoting social responsibility are inherently fit with the democratic and relational objectives of SEL (Jennings & Greenberg, 2009), which is also emphasized in the transformative SEL literature (Jagers et al., 2019). Placing these critical insights at the heart of the conceptual discussion serves an important purpose: it reframes SEL less as a toolkit for internal management and more as a deliberate project of creating classroom conditions in which agency is actively nurtured. From this standpoint, what we term participatory SEL ceases to appear as an optional or teacher-driven add-on. Instead, it emerges as a core, equity-aligned practice in which students are positioned as genuine co-authors of classroom culture, while teachers assume the role of facilitators who enable collective decision-making and shared construction of meaning.

In conclusion, these insights indicate that SEL identity encompasses more than mere knowledge and skills; it reflects a democratic approach to education. Aligning with this, equitable and civic-oriented SEL has been conceptualized (Owen & Phillips, 2025), in which SEC focuses on raising responsible citizenship. Teachers integrating SEL with an emphasis on social justice and community demonstrate the capacity of SEL to foster active citizenship (McGovern et al., 2023). Professional development should enhance teachers’ capacity to create participative, agency-promoting classroom practices, enabling them to perceive SEL as integral to their professional responsibility in fostering socially responsible and empowered students. Integrating SEL into teacher identity may mitigate criticisms of SEL as excessively technical or individualistic, reframing it as a foundation for relational, equitable, and civic-oriented education.

### 4.5. Managerial Expertise

The final component, managerial expertise, refers to teachers’ skills in resolving conflicts and managing the classroom effectively to maintain a positive, structured, and inclusive learning environment. A positive learning environment is characterized by the capacity to manage behavioral difficulties constructively while fostering a collaborative educational atmosphere. Classroom management contributes to self-efficacy in teacher identification, whereas conflict resolution is linked to relationship skills in SEL. Classroom management refers to teacher competence in organizing and structuring learning environments that are inclusive, productive, and emotionally safe (Pianta, 2001), whereas conflict resolution emphasizes teacher ability to mediate in ways that promote harmony and peaceful relations (CASEL, 2020). Based on the findings, managerial expertise comprised two methodologies.

The managerial expertise component of SEL-ID involves letting children solve conflicts and supporting children’s autonomy in resolving conflicts while intervening only when necessary to maintain safety and emotional well-being. The following is an example from one of the teachers in the study: *“I also like it when they realize that they can correct their mistakes”* (Beril, interview 2). We triangulated this data with classroom observation that mirrored the teacher’s belief in practice.

“A verbal argument broke out between two children. The teacher was standing nearby in the classroom. When the argument started, the teacher quietly watched them, occasionally making eye contact, while also observing the children playing in the classroom. The teacher did not intervene in the children’s verbal argument. One of the children asked his friend, “What happened, why do you feel that way?” The other child replied, “You used the bad word to me, it made me angry.” Then, one said, “I didn’t use that word to you; you misunderstood me,” and they resolved the argument between themselves and continued playing their game in the center.”

This was a kind of managerial expertise embedded not in control but in facilitation of social–emotional growth of children through teachers maintaining calm presence in the environment and providing agency to children to articulate their feelings on their own. SEL frameworks also show that effective conflict resolution improves students’ self-regulation and interpersonal abilities (Schonert-Reichl, 2017). Recent research indicates that classroom management based on social–emotional competence is a predictor of academic and socio-emotional results (Cipriano et al., 2023; McCormick et al., 2021).

Another example is from Selin, who emphasized the role of shared classroom rules as a guiding framework.

*“At the very simplest level, we are all familiar with our rules by now. Every day, based on my observations, I remind them of whatever they are most lacking in. Yes, this was in our class rules. Today, this was not really followed. I think some of our friends have forgotten these rules a little, so I remind them, saying, ‘Let’s all remember our rules together.”*

(Selin, interview 3.)

However, classroom observation of the same teacher revealed a more control-oriented approach. During one session, the teacher gathered the children around the table, instructed them to sit with their arms crossed, and called out individual names while reminding them that certain behaviors should not happen again. This discrepancy between verbal narrative and observed practice suggests that managerial expertise may vary between facilitative conflict resolution and directive behavior management. It also illustrates the tension between SEL-informed approaches and more traditional control-based classroom management, which is defined in previous teacher identity research (Hanna et al., 2020; Tschannen-Moran & Hoy, 2001). The identity in action tension can be interpreted through several contextual moderators. Firstly, teachers received traditional classroom management approaches, which depend on maintaining order in a behaviorist approach rooted in the 1960s (Emmer & Sabornie, 2014). Secondly, the Turkish ECE context is influenced by culturally embedded adult–child hierarchies. Teachers may position themselves as authoritative caregivers responsible for directing and protecting children (Gol-Guven, 2009). Finally, social desirability may lead teachers to articulate the ideals of “what a good” teacher should do. This tendency can create discrepancies between narrative and observation (Kaufman et al., 2016).

Within the teacher identity literature, classroom management is seen as part of self-efficacy, which is one’s ability to maintain order and control, and it focuses more on controlling disruptive behavior (Hanna et al., 2020; Tschannen-Moran & Hoy, 2001). Although teacher identity studies narrowly focus on behavior control, this study reframes this understanding with managerial expertise, which requires teachers’ relational facilitation in conflict resolution.

In sum, these results indicate that teachers perceive management expertise as a means of resolving conflict rather than imposing control, thereby placing themselves as facilitators of children’s problem-solving. The final component, managerial expertise, refers to teachers’ ability to mediate conflicts effectively and to manage the classroom in ways that create structure, inclusivity, and constructive responses to challenging behaviors.

## 5. Suggestions

This study embodies certain qualities that are best appreciated at a preconscious level, which may also inspire ideas for future research. First of all, the participants were 12 female teachers working in ECE centers within the same city. While this context provided rich and in-depth insights, examining SEL-ID across a wider range of geographical regions, institutional structures, and educational levels, including primary, secondary, and subject-specific branches, would offer valuable opportunities to understand how the construct manifests across diverse professional cultures. Including male teachers may also contribute to a more comprehensive portrait of the construct. Secondly, future studies may benefit from designing longitudinal or follow-up studies to explore the developmental nature of SEL-ID over time. Such studies could illuminate how SEL-ID evolves as teachers gain experience, engage in ongoing SEL-related practices, or participate in sustained professional learning, thereby revealing the dynamic and temporal aspects of the construct. Thirdly, future research may also investigate SEL-ID in educational settings where teacher–student interactions vary in intensity and structure. Comparing early childhood contexts with other branches of education could generate important insights into the contextual conditions that shape teachers’ SEL-related identity work.

Finally, there is promising potential in examining professional development models that explicitly aim to strengthen teachers’ SEL-ID. Future studies could explore how targeted PD designs influence teachers’ sense of identity, their SEC, and their classroom practices. Investigating how SEL components are introduced during pre-service education may further clarify how theoretical knowledge can be translated into practical strategies before teachers enter the profession (Matischek-Jauk & Reicher, 2021).

## 6. Implications

To support the effective integration of SEL-ID into teacher education and school systems, the implications summarized in Table 4 offer actions at both the individual (micro) and policy/program (macro) levels. For the self-perception component, teachers can engage in structured self-reflective activities that help them articulate professional values and growth areas, while at the macro-level, pre-service programs can involve reflective practice modules supported by mentors and guided observation protocols. Strengthening emotional literacy requires teachers to use emotion check-in routines and everyday regulation strategies, whereas teacher education curricula should systematically embed emotion regulation training. In addition, schools can implement well-being policies that normalize emotional expression and support.

Within interpersonal relations, micro-level implications include strengthening parent–teacher partnerships. Also, teachers can be encouraged to engage in peer feedback cycles to cultivate professional trust. At the macro-level, school-wide restorative communication policies and efforts to build a relational workplace culture can enhance collegial collaboration and emotional safety among staff. The participatory SEL component highlights the value of daily routines that encourage children’s involvement in decision-making. Considering this finding, school and district policies can promote participatory classroom cultures by valuing student agency and shared decision-making in curriculum and routines.

Finally, managerial expertise requires teachers to model and teach conflict resolution explicitly and to design individualized support plans for children who need targeted guidance. At the macro-level, SEL-aligned classroom management training can ensure coherence between teacher preparation and school practices, reframing management as a developmental and relational process rather than a compliance-oriented task. Collectively, these implications translate the SEL-ID framework into actionable guidance that bridges teacher beliefs, classroom practice, and institutional support structures.

## 7. Conclusions

This study explored and conceptualized SEL-ID as a novel construct using teacher identity as an analytical lens to illuminate the missing identity-related dimension of SEC, teachers’ ownership of SEL, which helps explain why technical knowledge and skills alone are insufficient to translate SEC into effective SEL practices. The findings enhance current theories by employing five interrelated components to define SEL-ID: self-perception, emotional literacy, interpersonal relations, participatory SEL, and managerial expertise. This study highlights how SEL evolves into a component of teacher identities, influencing their self-perception, student interactions, and classroom planning, rather than merely being regarded as a technical skill set. SEL-ID offers a new perspective on the reasons why a grasp of SEL does not consistently emerge in classroom practices. Teachers’ SEL identity exhibits substantial similarities with overall teacher identity, encompassing motivation, self-perception, job satisfaction, task interpretation, commitment, experiential background, and self-efficacy. Comprehending these relationships is crucial for contextualizing SEL-ID within the current academic discourse on teacher professional identity. SEL-ID is not a standalone construct but is intricately connected to fundamental elements of teacher identity. Motivation, self-perception, work satisfaction, lived experiences, self-efficacy, and task perception collectively influence teachers’ implementation of SEL in their classrooms. This convergence highlights the significance of analyzing SEL-ID as both a separate and cohesive aspect of teacher identity. The SEL-ID components offer a comprehensive insight into the methods by which teachers adopt and exemplify SEL. Furthermore, SEL identity is flexible, similar to teacher identity; it develops over time and can be seeded during pre-service teacher education and then strengthened through reflective learning and professional experiences. Professional development programs should provide space for teachers to explore themselves through reflective practices, enable teachers to play a role in collaborative discussions, and support their capacity related to SEL-ID.

The findings also align with the concepts highlighted in transformative SEL. The participatory practices identified in SEL-ID, including children’s involvement in decision-making, correspond with transformative SEL’s emphasis on agency and the role of students as active participants in classroom culture. In the same way, the focus that teachers put on collegial support and relational trust in interpersonal relations components shows a sense of belonging that is an important concept in transformative SEL. Additionally, the incorporation of SEL into educators’ values, commitments, and professional objectives illustrates the identity-driven essence of SEL-ID, emphasizing that effective SEL arises not solely from technical proficiency but from teachers’ self-perception.

One of the noteworthy findings of the study is the identification of self-efficacy as a cross-cutting construct that emerges across the identified components. Specifically, student engagement efficacy was embedded in participatory SEL, where teachers’ confidence in fostering student voice and active participation was evident. Classroom management efficacy was reflected in managerial expertise, associated with teachers’ capacity to resolve conflict or facilitate it constructively. Within emotional literacy, teachers demonstrated emotional self-efficacy, which refers to their perceived ability to regulate their own emotions and respond sensitively to students. Finally, collective efficacy was located within interpersonal relations, showing teachers’ confidence in their colleagues’ and the school community’s capacity to provide mutual support. This distribution aligns with multidimensional models of teacher self-efficacy (Goddard et al., 2000; Hanna et al., 2019; Skaalvik & Skaalvik, 2007; Tschannen-Moran & Hoy, 2001) and supports expanded conceptualizations that extend beyond task-related efficacy (e.g., emotional self-efficacy, Hen & Goroshit, 2016; Kirk et al., 2008). Supporting the complexity of self-efficacy, the TALIS 2024 report conceptualizes it as a multidimensional construct embedded within teachers’ professional competence, teamwork, and emotional resources rather than as an isolated factor (OECD, 2025). This can be interpreted as self-efficacy being distributed across teacher identity rather than being a separate trait. Along with the present finding and conceptual framework by TALIS 2024 report, SEL-ID provides efficacy domains while also offering evidence for the relational and embedded nature of the concept. From this perspective, self-efficacy in SEL-ID is not singular but emerges in differentiated forms across the relational, emotional, participatory, and managerial domains of teaching, which future studies could examine in greater depth.

## Figures and Tables

**Table 1:** The demographics of teachers.

Pseudonyms	Education Level	Experience
Buse	Bachelor’s degree	0–3 years
Deniz	Bachelor’s degree	0–3 years
Selin	Bachelor’s degree	0–3 years
Seda	2-year degree	4–6 years
Senem	Bachelor’s degree	0–3 years
Ayla	Bachelor’s degree	4–6 years
Beril	Bachelor’s degree	4–6 years
Hale	Bachelor’s degree	4–6 years
Sara	Bachelor’s degree	4–6 years
Tansu	2-year degree	7–9 years
Gonca	Bachelor’s degree	10–12 years
Ada	Master’s degree	16 years and above

**Table 2:** Data collection method, frequency and duration, and examples of interview questions.

Data Collection Methods	Frequency & Duration	Interview Questions
Semi-structured interviews	3 interviews per teacher (~45 min each)	1st: Teacher narratives and role (“How did you decide to become a teacher?”) 2nd: Emotions and SEL beliefs (“How do your emotions play a role in your teaching?”) 3rd: Classroom priorities and SEL strategies (“What are your priorities in the classroom?”)
Classroom observations	3 observations per teacher (each ~half day)	Guided by CLASS, we focused on teacher sensitivity, teachers’ emotional expressiveness, teacher–child interactions, emotional climate, and SEL integration

**Table 3:** The examples for coding.

Domain and Definition	Axial Coding	Open Code	Illustrative Quotations
Interpersonal relations	Parent–Teacher Partnership	Seeking parental support for problem behavior	“Some gave up, and some reacted. I told them, ‘Don’t give up, we’ll keep going…’ We were constantly working together.” Teachers motivate parents to remain engaged, even when they feel overwhelmed (relational perseverance).
Trust-based, restorative, and solution-focused relationships with parents, and professional relationships with colleagues that are open, constructive, respectful, and emotionally supportive		Professional relations with parents	“If the crisis is my fault, I apologize… We can talk to families about the situation and exchange ideas on how to fix it.” Accepting mistakes, modeling accountability, and using dialogue as a restorative tool (interpersonal responsibility).
	Collaborative Colleague Dynamics	Being open for feedback	“I really want someone to evaluate it… Please tell me if you think I’ve done something wrong.” Desire for honest, improvement-oriented feedback; evidence of relational trust.
		Open communication with colleagues	“In calmer times… we talk like this: ‘This is how I understood it, this is how I felt… I wish you hadn’t said those things, you could have said it differently.’ With open communication, we clarify any misunderstandings and focus on resolving them. Ultimately, whoever is right apologizes.” Using “I” language, expressing feelings calmly, clarifying misunderstandings, focusing on resolution rather than being right, and apologizing when necessary, which reflects mature, restorative, and emotionally safe communication among colleagues.
		Collaborative teamwork and mutual respect	“If there’s a problem, everyone covers each other’s backs immediately.” Emotional security, collective care, and mutual respect are emotional resources.
		Supportive colleague relationship	Coworkers act as “emotional buffers, sources of professional learning, and co-creators of a positive school climate.” Mutual support helps teachers manage stress and resolve challenges.

**Table 4:** The micro- and macro-level integration of SEL-ID.

SEL-ID Component	Micro-Level (Teacher and Classroom Level)	Macro-Level (Policy and Program Design-Based)
Self-perception	Self-reflective activities	Structured reflective practice modules in pre-service programs
Emotional Literacy	Engaging emotion check-in routines	PD curricula embed emotion regulation training Schools establish well-being policies
Interpersonal Relations	Strengthen parent–teacher partnerships Engagement in peer feedback cycles	Implementation of school-wide restorative communication policies Building a relational school culture among colleagues
Participatory SEL	Daily routines encouraging children’s involvement	School policies that support a participatory classroom culture
Managerial Expertise	Teaching and modeling conflict resolution in the classroom in an explicit manner throughout the process Individualized support plans for children having difficulty in resolving conflicts	SEL-aligned classroom management training

## Data Availability

The data supporting the findings of this study are not publicly available in order to protect participant confidentiality and due to ethical restrictions associated with the informed consent process.
